# Sensorimotor Robotic Measures of tDCS- and HD-tDCS-Enhanced Motor Learning in Children

**DOI:** 10.1155/2018/5317405

**Published:** 2018-12-18

**Authors:** Lauran Cole, Sean P. Dukelow, Adrianna Giuffre, Alberto Nettel-Aguirre, Megan J. Metzler, Adam Kirton

**Affiliations:** ^1^Department of Neurosciences, University of Calgary, 3330 Hospital Drive NW, Calgary, AB T2N 4N1, Canada; ^2^Hotchkiss Brain Institute, University of Calgary, 3330 Hospital Drive NW, Calgary, AB T2N 4N1, Canada; ^3^Cumming School of Medicine, University of Calgary, 3330 Hospital Drive NW, Calgary, AB T2N 4N1, Canada; ^4^Department of Clinical Neurosciences, University of Calgary, 3330 Hospital Drive NW, Calgary, AB T2N 4N1, Canada; ^5^Department of Pediatrics, University of Calgary, 2888 Shaganappi Trail NW, Calgary, AB T3B 6A8, Canada; ^6^Department of Community Health Sciences, University of Calgary, 2500 University Drive NW, Calgary, AB, T2N 1N4, Canada; ^7^Alberta Children's Hospital Research Institute, 2888 Shaganappi Trail NW, Calgary, AB T3B 6A8, Canada

## Abstract

Transcranial direct-current stimulation (tDCS) enhances motor learning in adults. We have demonstrated that anodal tDCS and high-definition (HD) tDCS of the motor cortex can enhance motor skill acquisition in children, but behavioral mechanisms remain unknown. Robotics can objectively quantify complex sensorimotor functions to better understand mechanisms of motor learning. We aimed to characterize changes in sensorimotor function induced by tDCS and HD-tDCS paired motor learning in children within an interventional trial. Healthy, right-handed children (12–18 y) were randomized to anodal tDCS, HD-tDCS, or sham targeting the right primary motor cortex during left-hand Purdue pegboard test (PPT) training over five consecutive days. A KINARM robotic protocol quantifying proprioception, kinesthesia, visually guided reaching, and an object hit task was completed at baseline, posttraining, and six weeks later. Effects of the treatment group and training on changes in sensorimotor parameters were explored. Twenty-four children (median 15.5 years, 52% female) completed all measures. Compared to sham, both tDCS and HD-tDCS demonstrated enhanced motor learning with medium effect sizes. At baseline, multiple KINARM measures correlated with PPT performance. Following training, visually guided reaching in all groups was faster and required less corrective movements in the trained arm (*H*(2) = 9.250, *p* = 0.010). Aspects of kinesthesia including initial direction error improved across groups with sustained effects at follow-up (*H*(2) = 9.000, *p* = 0.011). No changes with training or stimulation were observed for position sense. For the object hit task, the HD-tDCS group moved more quickly with the right hand compared to sham at posttraining (*χ* ^2^(2) = 6.255, *p* = 0.044). Robotics can quantify complex sensorimotor function within neuromodulator motor learning trials in children. Correlations with PPT performance suggest that KINARM metrics can assess motor learning effects. Understanding how tDCS and HD-tDCS enhance motor learning may be improved with robotic outcomes though specific mechanisms remain to be defined. Exploring mechanisms of neuromodulation may advance therapeutic approaches in children with cerebral palsy and other disabilities.

## 1. Introduction

Transcranial direct-current stimulation (tDCS) is a form of noninvasive brain stimulation that can modulate cortical excitability with associated behavioral changes [1]. Conventional tDCS has traditionally been applied using two large sponge electrodes (1 × 1 tDCS), inducing broad electric fields between the anode and cathode. More recently, modified montages have created options for high-definition tDCS (HD-tDCS) with more focal application of current to targeted cortical areas. Such montages may involve a central anode surrounded by 4 cathodes (4 × 1 HD-tDCS). There are many reasons to suspect that tDCS effects differ in the developing brain including current modeling investigations that suggest that more intense and diffuse electric fields are induced by tDCS in children [2]. Therefore, there is a need to investigate tDCS applications and mechanisms in the developing brain.

One of the most well-studied effects of tDCS is its ability to enhance motor learning [3]. When paired with training of the contralateral hand, anodal tDCS centered on the primary motor cortex may improve motor acquisition and retention of skill. We have demonstrated in healthy school-age children that M1-targetted tDCS over three consecutive days of training enhances motor learning as assessed by improvements on the Purdue pegboard test (PPT) [4]. These improvements were retained six weeks later. HD-tDCS has not been commonly applied in the context of motor learning, but one adult study suggests that M1 HD-tDCS may increase visuomotor adaptation assessed through tracing time and accuracy in several mirror drawing tasks within a single session [5]. Bihemispheric M1 HD-tDCS paired with unimanual and bimanual motor training for three consecutive days also showed improvements in bimanual hand dexterity [6]. Recently, we described similar effects of both anodal 1 × 1 tDCS and high-definition tDCS (HD-tDCS) motor cortex stimulation over five days of training in healthy school-age children [7].

Importantly, understanding the mechanisms that underlie tDCS modulation is limited [8] and virtually unstudied in the developing brains of children. While progress continues at the preclinical, cellular, imaging, and other systems level approaches, few studies have examined the behavioral mechanisms by which tDCS might enhance motor learning [9–12]. Reaching is a crucial function for everyday life that requires intricate integration between motor and sensory systems. Strong connections are evident between M1 and the somatosensory cortex, and the stimulation of M1 may impact somatosensory processing [13]. The integrity of M1 and somatosensory cortex connections have also been previously correlated with PPT scores [14]. Studies of the effects of motor learning on sensory function suggest direct interactions between M1 and the primary sensory cortex [15]. Training on motor tasks not only improves motor performance but may also enhance sensory function [16]. Position sense is independent of reaching task performance; however, its integration is functionally relevant for therapeutic applications [17]. Proprioception is a composite of position sense, which refers to the static sense of limb position, and kinesthesia, the dynamic sense of limb motion [18–20]. Functional proprioception is vital in providing feedback required for motor control, coordination, and learning. How all of these elements change during motor learning and neuromodulation is unknown in children.

The question of how sensorimotor performance changes with learning and interventions like brain stimulation can be more accurately investigated using robotics. Robotic tools have been used extensively to examine motor learning for the last few decades [21, 22]. Robotic tools improve on many of the observer-based clinical tools that are historically utilized to quantify sensory and motor functions. Most of these clinical tools have shortcomings, including a lack of sensitivity to small changes in function and poor interrater reliability [23–25]. Members of our team have helped develop more accurate, objective, and reliable measures of upper limb sensory and motor functions using robotic technology [23, 26–30]. We have used this technology to study healthy adults and clinical populations and, more recently, to quantify sensorimotor function in children [31–33]. KINARM measures have been correlated with evidence-based functional outcomes and imaging biomarkers, confirming clinical relevance [33]. The KINARM provides a unique opportunity to quantify complex changes in sensorimotor function during motor learning and its neuromodulation. Such studies serve to investigate the mechanisms of motor learning, as well as increase our understanding of how tDCS influences motor and sensory functions in the developing brain.

An improved understanding of motor learning neuromodulation mechanisms has immediate translational relevance for clinical populations. For example, perinatal stroke is the leading cause of hemiparetic cerebral palsy [34] where sensorimotor dysfunction results in lifelong disability [31, 35]. Robotic measures of visually guided reaching, kinesthesia, and position sense in affected children have defined mechanisms of disability, imaging biomarkers, and novel therapeutic targets [31–33, 36]. The improved models of sensorimotor development that result from these studies inform novel targets for neuromodulation including translation into multiple recent controlled trials [37, 38]. The ability to measure detailed sensorimotor functions with robotics before and after such interventions has the potential to further inform mechanisms of interventional plasticity.

Here, we aimed to characterize sensorimotor changes within a blinded, controlled interventional trial of tDCS- and HD-tDCS-enhanced motor training in healthy children. We hypothesized that both motor and sensory measures would change with motor learning, with tDCS specifically conferring an improvement in position sense.

## 2. Materials and Methods

### 2.1. Participants

Participants (age 12–18 years old) were recruited from our population-based Healthy Infant and Children Clinical Research Program (HICCUP) and from the community. Inclusion criteria were (1) typical neurodevelopment, (2) right-handed (modified Edinburgh handedness inventory was applied at enrollment to confirm a laterality index of ≥−28), and (3) healthy (no major medical condition). Exclusion criteria were (1) neuroactive medications or (2) noninvasive brain stimulation [39] or MRI contraindications. Participants and their guardians self-reported no neuropsychological, developmental diagnosis, or neuroactive medications. The Research Ethics Board at the University of Calgary approved all experimental procedures. Participants or their guardians provided written informed consent, and when necessary, participants' assent was obtained.

### 2.2. Study Design

Accelerated motor learning in pediatrics (AMPED) was a double-blind, randomized, sham-controlled interventional trial to determine the effects of tDCS and HD-tDCS on motor learning in children (ClinicalTrials.gov: NCT03193580). Participants were computer randomized into one of the three intervention groups: (1) sham, (2) right (contralateral to the trained hand) hemisphere 1 mA anodal tDCS (tDCS), or (3) right (contralateral) hemisphere 1 mA anodal HD-tDCS (HD-tDCS). Participants completed a series of assessments, including KINARM measures (see below) at baseline (pretraining), posttraining, and at six-week follow-up (follow-up). Training consisted of five consecutive days of left-hand training paired with stimulation. Details of the protocol are described elsewhere [7] and summarized in the Supplementary Figure 1.

### 2.3. Motor Training and Transcranial Direct-Current Stimulation

Participants trained their left, nondominant hand on the Purdue pegboard test (PPT_L_) for 20 minutes for five consecutive days. The PPT is a validated measure of hand dexterity [40]. Participants have 30 seconds to place as many pegs as they can with their left hand (PPT_L_). The average total number of pegs placed over three trials was scored. The nondominant hand was used to assess motor learning to achieve a steeper learning curve and avoid a possible “skill ceiling.” The PPT_L_ was performed prior to stimulation each day. After stimulation began, the PPT_L_ was completed 5, 10, and 15 minutes into the stimulation period, and again after stimulation ended (three repetitions per time point). The same training was repeated on days 2 to 5. After the final training block on day 5, participants underwent the same series of assessments as day 1. Participants returned six weeks later and repeated all assessments to examine retention of acquired motor skills.

Noninvasive brain stimulation was administered by experienced personnel according to established tDCS methods in adult and pediatric populations [4, 9, 41]. Participants' “hotspot” (region of the M1 that evoked maximum response of the first dorsal interosseous muscle) was identified using transcranial magnetic stimulation (Magstim, Cardiff, UK, and Axilum Robotics, Strasbourg, France). This location was coregistered to each participants' neuroanatomical T1 MRI and marked on the scalp. The electrode montages are demonstrated in Supplementary Figure 2. Participants randomized to tDCS or sham had two square saline-soaked sponge electrodes (25 cm^2^ EasyPads, Soterix Medical Inc., NY, USA) placed on the scalp and held in place by a commercially available light plastic headband sized for children (SNAPstrap, Soterix Medical Inc., NY, USA). The anode was centered over the “hotspot” with the cathode over the contralateral supraorbital area. Electric current was applied using a Soterix 1 × 1 stimulator (Soterix Medical Inc., NY, USA). For participants randomized to the HD-tDCS group, an EEG cap with a ring of four cathodes surrounding a single anode (SmartScan stimulator, HD-tDCS adaptor, 1 cm diameter circular electrodes, electrode holder, and gel; Soterix Medical Inc., NY, USA) was placed over the right M1. Electrodes were connected to a 1 × 1 stimulator attached to a 4 × 1 HD-tDCS adaptor (Soterix Medical Inc.). In all conditions, the strength of current applied was 1 mA. For participants receiving tDCS or HD-tDCS, the current was ramped up from 0 to 1 mA over 30 seconds, held at 1 mA for 20 minutes, and then ramped down to 0 mA over 30 seconds. For participants randomized to sham, current was also ramped up from 0 to 1 mA over 30 seconds and then immediately ramped down to 0 mA. This sham protocol produces similar sensations to active forms of tDCS; however, it does not induce any lasting changes in cortical excitability [42]. At the end of each session (days 1–5), participants completed a pediatric noninvasive brain stimulation safety and tolerability questionnaire [43].

### 2.4. Robotic Assessment of Sensorimotor Function

**Figure 1:**
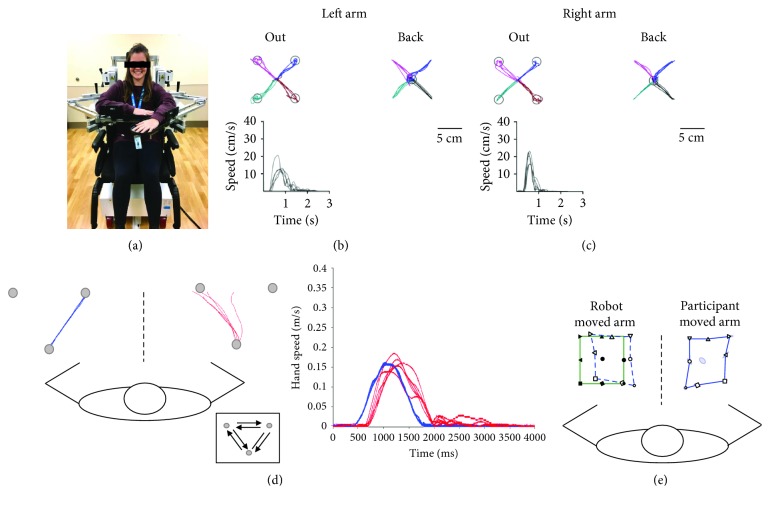
Pediatric KINARM robot tasks for visually guided reaching, kinesthesia, and position matching tasks for an exemplar 18-year-old female. (a) Frontal view of the KINARM robotic apparatus. (b) Visually guided reaching performance for the left hand and speed profile for the movement out from the center to the bottom left target. (c) Visually guided reaching performance for the right hand and speed profile for the movement from the center out to the bottom left target. The participant reached out from a central target to one of the four peripheral targets and reached back to the center target. (d) Kinesthesia single direction hand paths. Blue line represents the robot movement of the passive left arm; red lines represent the active arm path. Grey circles represent the location of robotic movement targets. Hand speed profile shows the speed of the passive (blue) and active (red) arms. (e) End positions for the position matching task. Closed symbols represent the positions where the moved the participants' passive left arm. The solid green lines represent the border of the outer 8 targets. Open symbols on the right represent where the participant mirror matched with their active right arm. The ellipses represent variability of matching (1SD). Open symbols on the left are the mirrored representation of the subject's attempts to match so that the readers can easily compare the two arms.

The primary outcomes of the current study were derived from a standardized assessment of sensorimotor function using the KINARM robot. Robotic assessments were performed at Alberta Children's Hospital using the KINARM robotic exoskeleton (BKIN Technologies Ltd., Kingston, Ontario, Canada) to measure a variety of sensorimotor tasks as previously described in healthy and stroke-affected adults [26–28] and children [31, 32]. Participants sat in a modified wheelchair base with each arm supported in the horizontal plane by the exoskeleton [44]. To achieve comparable arm positioning for smaller children, modifications were made by adding up to 5 cm of padding under the seat cushion. After the participant was set-up in the KINARM, they were wheeled into an augmented reality workstation where virtual targets were projected through a semitransparent screen (see Figure 1). Four tasks were completed in a standardized sequence.

#### 2.4.1. Visually Guided Reaching

In a unimanual visually guided reaching task [17, 27], participants were instructed to reach as quickly and accurately from a fixed central target (2 cm diameter) to one of the four peripheral circular targets (2 cm diameter) located 6 cm away. The robot did not assist or provide resistance. Participants first completed the task with their dominant arm, followed by the nondominant arm. We analyzed performance in the nondominant arm. A total of 20 reaches were completed with each arm (five reaches per target). Targets were presented in a pseudorandomized order. The task was identical to previous work and used the same metrics described elsewhere [33]. Performance was quantified by six parameters:

1. Postural hand speed: a measure of upper limb postural control while trying to hold at the center target, measured by mean hand speed for 500 ms before the peripheral target appears (cm/s)
2. Reaction time: the time between appearance of peripheral target and onset of movement (seconds)
3. Initial direction error (IDE): the angular deviation between a straight line from the central target to peripheral target and the actual path taken in the initial phase of movement (degrees)
4. Number of speed peaks per movement (NSP): the number of hand speed maxima between movement onset and movement offset
5. Total movement time (MT): the total movement time measured in seconds
6. Maximum hand speed: the maximum speed reached in the task (cm/s)

#### 2.4.2. Kinesthesia Task

The kinesthesia task assessed participants' sense of upper limb motion. With vision of the arms occluded, the participants' arms were brought into a mirrored starting position at one of the three possible positions. To do this, the robot moved the nondominant arm passively to a position and the subject then placed the index finger of their opposite active arm, represented by a white circle in the virtual environment, into a red target presented in a mirrored position in the workspace. When the subject placed the white circle in the red target, a trial was initiated [26]. The target then disappeared from the workspace, and the robot initiated the movement of the nondominant (passive) arm at 0.18 m/s for 12 cm to one of the other 2 targets in the workspace. As soon as participants felt the initiation of movement, they were asked to mirror match the speed, amplitude, and direction of movement with their dominant (active) arm. The order of targets was pseudorandomized, and participants completed six blocks of six trials. The task was completed with vision of the arms occluded. The task was identical to the previous work [32], and the parameters have been thoroughly described elsewhere [26, 32, 45]. Task performance was measured by the following parameters:

1. Response latency (RL): the time to initiate a matching movement in response to the robotic movement (milliseconds)
2. Initial direction error (IDE): the angular deviation from the direction of the robotic movement and active arm length (degrees)
3. Peak speed ratio (PSR): the ratio of how well the participant matched the peak speed of the active arm to that of the robotic arm. Ratios < 1 indicate movement slower than the robot; ratios > 1 indicates faster than the robot
4. Path length ratio (PLR): the ratio of the length of active arm movement relative to the length of the robot-moved passive arm

The standard deviations across all movements were also classified as the variability of each parameter: RLv, IDEv, PSRv, and PLRv.

#### 2.4.3. Position Matching Task

The arm position matching task measured position sense ability [28]. With bell-shaped velocity, the robot moved the participants' nondominant (passive) arm to one of the nine spatial targets, each separated by 6 cm. When the movement was complete, participants were instructed to match their dominant (active) arm to the mirror image location of the passive arm. A total of 54 movements were performed involving six blocks of trials where the targets were pseudorandomized. This task was identical to the previous work [31], and the parameters are described elsewhere [28, 31]. The task was completed with vision occluded. The performance was quantified by three parameters:

1. Variability (Var_xy_): endpoint variability (mean standard deviation) of the active arm position in the matched location, measured in centimeters
2. Contraction/expansion: the ratio of the area moved over by active hand relative to the area moved over by the passive hand (values < 1 demonstrate contraction)
3. Systematic shift (Shift_xy_): the spatial translation of the workspace between the passive and active hands, measured in centimeters

#### 2.4.4. Object Hit Task

The KINARM object hit task assessed rapid visuomotor function, decision-making skills, and bilateral motor control [46]. Virtual balls fell from the top of the workspace towards the participant who used 5 cm virtual paddles located at their hands to hit the balls away. A total of 300 balls fell from 10 bins separated equally across the top of the workspace [47]. As the task continued, the difficulty increased, where balls fell at greater speeds and appeared more often. Twelve parameters were collected as previously described [46]. A learning effect has been observed in children, and this effect was diminished after performing the task twice [48]. Therefore, a practice effect trial was completed at the beginning of each assessment. Performance was quantified with seven variables:

1. Total balls hit: total number of balls successfully hit throughout task
2. Total balls with left or right hand: total balls hit with either left or right hand
3. Median error: the percentage of the task that is complete at the time subjects make 50% of their errors (percentage)
4. Mean hand speed for the right and left hands: the average hand speed for each hand (cm/s)
5. Hand bias hits: quantifies hand dominance in balls hit. Calculated as (Total right hand hits − Total left hand hits)/(Total right hand hits + Total left hand hits)
6. Hand movement bias area: quantifies differences in size of workspace of each hand
7. Hand bias speed: quantifies the difference between mean hand speeds of the left and right hand

### 2.5. Statistical Analysis

Nonparametric statistics were used to examine differences in KINARM task scores due to relatively small sample size and the lack of knowledge on whether the true distribution of the measurements was normal. Shapiro-Wilk tests determined the normality of the sampled data distributions. Spearman correlation was used to identify associations between baseline robotic scores, PPT performance, and age. Kruskal-Wallis one-way analysis of variance on ranks was used to examine possible differences in baseline scores across intervention groups and examine intervention effects at each time point. Post hoc analysis employed by Dunn's test. One-way analyses of variance (ANOVA) and chi-square/Fischer exact tests compared group demographics and baseline PPT_L_ score. Paired *t*-tests examined the differences in the left- and right-hand baseline PPT scores. Significance values were adjusted for multiple comparisons using Bonferroni corrections. The Friedman test was used to explore training effects within and across intervention groups with post hoc Wilcoxon signed-rank test analysis performed using SigmaPlot 12.5 (Systat Software Inc., San Jose, USA) and SPSS (IBM, Armonk, NY, USA).

## 3. Results

### 3.1. Population Characteristics

Twenty-four children were recruited and completed all training and robotic measures (median age 15.5 years, range 12–18 years, 52% female). Age, sex distribution, self-reported handedness, and baseline clinical function measures did not differ between intervention groups (*p* > 0.323). All groups demonstrated a higher PPT_R_ compared to PPT_L_ scores (*p* < 0.001). Baseline KINARM robotic scores did not differ between groups (*p* > 0.05). Population characteristics by the intervention group are summarized in Table 1.

### 3.2. Motor Learning

The effects of intervention on motor learning were described in detail elsewhere [7]. In summary, all participants demonstrated an increased number of pegs placed over the five days of training on the primary training task (PPT_L_), regardless of the intervention group (*p* < 0.001). Participants receiving tDCS or HD-tDCS had significantly enhanced rates of learning compared to sham (tDCS *t*(117) = 2.058, *p* = 0.042; HD-tDCS *t*(117) = 1.986, *p* = 0.049) with moderate to large effect sizes (Cohen's *d* tDCS = 0.655, HD-tDCS = 0.851) and sustained effects at 6 weeks.

### 3.3. Visually Guided Reaching

**Figure 2:**
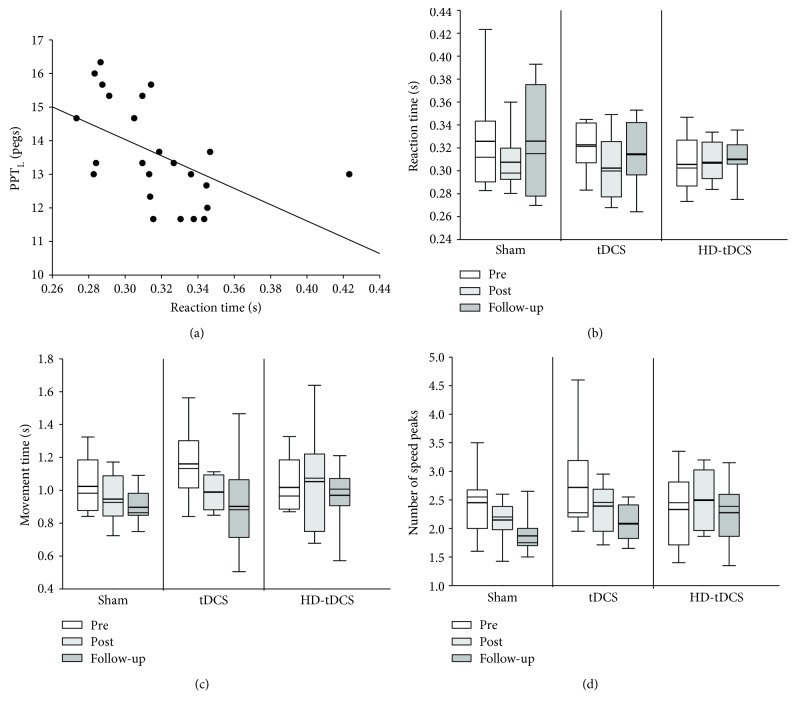
Visually guided reaching with the left hand. (a) Scatter plot of the baseline left-hand Purdue pegboard test (PPT_L_) score and baseline reaction time. (b) Reaction time at pretraining (white), posttraining (light grey), and follow-up (dark grey) across the three intervention groups: sham, tDCS, and HD-tDCS. (c) Total movement time across the three intervention groups at pretraining, posttraining, and follow-up. (d) The number of speed peaks across the three intervention groups at pretraining, posttraining, and follow-up.

Outcomes for left hand visually guided reaching are summarized in Figure 2. At baseline, no associations were observed with age in any of the six robotic parameters (all *r* < 0.209, all *p* > 0.072). Baseline left hand visually guided reaching reaction time was negatively correlated with the baseline PPT_L_ score (*r* = −0.582, *p* = 0.003) (Figure 2(a)), where quicker reaction times correlated with a higher PPT_L_ score. Reaction time in the left hand was not significantly altered by training or intervention (both factors *H*(2) < 3.083, *p* > 0.214) (Figure 2(b)). Movement time with the left hand across all groups decreased over the training period (*H*(2) = 9.250, *p* = 0.010) (Figure 2(c)), where post hoc comparisons identified a significant reduction from pretraining to follow-up (*χ* ^2^(2) = 3.031, *p* = 0.007). There was also an overall significant effect of training in the number of speed peaks (*H*(2) = 6.333, *p* = 0.042) (Figure 2(d)), meaning subjects tended to make less submovements during left-hand reaching. Post hoc comparisons identified a marginal reduction from pretraining to follow-up (*χ* ^2^(2) = 2.309, *p* = 0.063). The left hand visually guided reaching initial direction error, postural speed, and left-hand maximum speed were not significantly affected by the training or intervention group (all *p* > 0.05).

### 3.4. Kinesthesia

**Figure 3:**
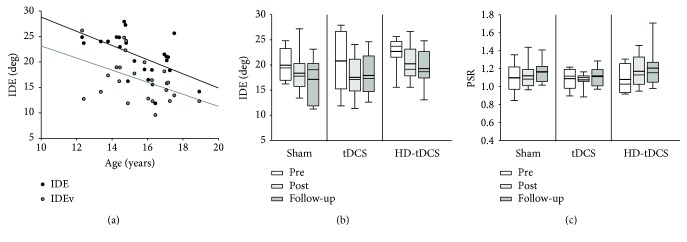
Kinesthesia task. (a) Scatter plot of age versus baseline initial direction error (IDE) (black circles) and variability of IDE (IDEv) (grey circles). (b) The baseline IDE across the three intervention groups at pretraining (white), posttraining (light grey), and follow-up (dark grey). (c) The peak speed ratio (PSR) across the pretraining, posttraining, and follow-up in the three intervention groups.

Figure 3 summarizes the kinesthesia outcomes. Baseline IDE (*r* = −0.544, *p* = 0.006) and IDEv (*r* = −0.461, *p* = −0.024) were correlated with age (Figure 3(a)). Baseline PSRv showed a weak correlation with age (*r* = −0.399, *p* = 0.053). There was a significant effect of training on IDE (*H*(2) = 9.000, *p* = 0.011), where post hoc comparisons revealed a decrease in IDE between pretraining vs. posttraining (*χ* ^2^(2) = 2.598, *p* = 0.028) and pretraining vs. follow-up (*χ* ^2^(2) = 2.598, *p* = 0.028) (Figure 3(b)). Thus, the ability to mirror match the direction of movement with the untrained hand in the kinesthesia task improved with training across all groups. There was no significant effect of training on IDEv (*χ* ^2^(2) = 4.750, *p* = 0.093) (Figure 3(a)). There was an overall marginal training effect seen in PSR (*H*(2) = 4.750, *p* = 0.093) (Figure 3(c)). There were no differences in PSR between intervention groups (*H*(2) < 1.221, *p* > 0.542). There was also no interventional (all *H*(2) < 3.141, *p* > 0.207) or time (*H*(2) = 2.083, *p* = 0.353) effects on PLR. However, there was an overall effect of training within the HD-tDCS group (*χ* ^2^(2) = 10.750, *p* = 0.005), with a significant increase in PLR from baseline to follow-up (*χ* ^2^(2) = 3.250, *p* = 0.003). There was no effect of training or intervention on response latency (both *p* > 0.05).

### 3.5. Position Matching

**Figure 4:**
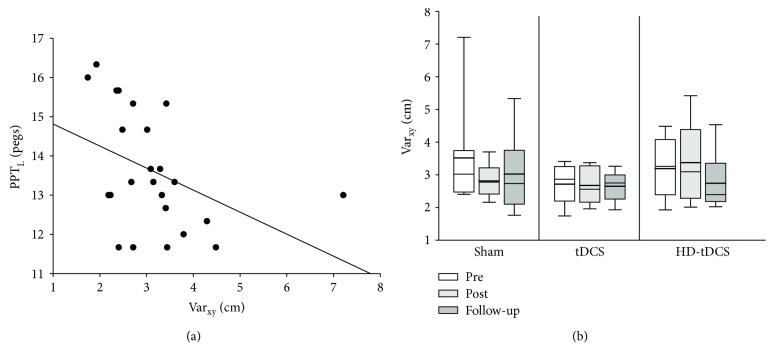
Position matching task endpoint variability (Var_xy_). (a) Scatter plot of baseline Var_xy_ and the baseline left-hand Purdue pegboard test (PPT_L_) score. The Var_xy_ across three intervention groups at pretraining (white), posttraining (light grey), and follow-up (dark grey).

Outcomes for the position matching task are summarized in Figure 4. None of the three measures of Var_xy_, Shift_xy_, or contraction/expansion correlated with age (all *r* > 0.290, *p* > 0.077). The primary position matching outcome of Var_xy_ correlated with baseline PPT_L_ (*r* = −0.540, *p* = 0.006), where lower variability was correlated with higher (better) PPT_L_ scores (Figure 4(a)). Var_xy_ performance did not improve with training (*H*(2) = 0.750, *p* = 0.687) (Figure 4(b)). There was no significant effect of the training or intervention group on Var_xy_, Shift_xy_, or contraction/expansion (all *p* > 0.05).

### 3.6. Object Hit

**Figure 5:**
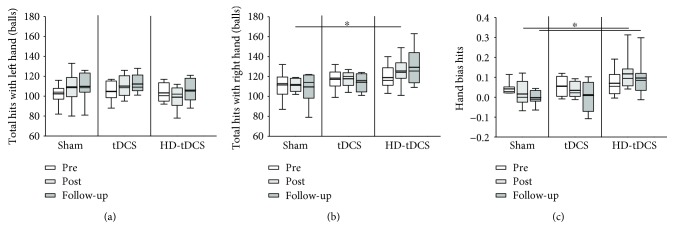
Object hit task. (a) The number of total hits with the left hand and (b) number of total hits with the right hand across the three intervention groups at pretraining (white), posttraining (light), and follow-up. (c) The hand bias hits across the three intervention groups at pretraining, posttraining, and follow-up ^∗^ *p* < 0.05.

Figure 5 summarizes the outcomes for the object hit task. There was a significant correlation between baseline total hits and age (*r* = 0.428, *p* = 0.037) where older participants hit more balls. There was a modest correlation between the baseline PPT_L_ score and total hits (*r* = 0.385, *p* = 0.062), where higher PPT_L_ scores correlated with more objects hit. There was no significant effect of the training or intervention group on total balls hit (all *p* > 0.05). There was an overall training effect on the number of balls hit with the left hand (*H*(2) = 6.404, *p* = 0.041) (Figure 5(a)). However, post hoc comparisons did not demonstrate a difference between time points. A Benjamini-Hochberg procedure was performed to further examine post hoc comparisons, and there was a significant difference between pretraining and follow-up (*p* = 0.017) and posttraining compared to follow-up (*p* = 0.033). Baseline total hits with the right hand were correlated with age (*r* = 0.410, *p* = 0.046). There was no training effect on number of balls hit with the right hand (*H*(2) = 4.067, *p* = 0.131) (Figure 5(b)). A significant intervention effect at posttraining was identified (*H*(2) = 6.563, *p* = 0.038) where post hoc analysis suggested that the HD-tDCS group hit more balls with the right hand at posttraining compared to sham (*χ* ^2^(2) = 2.550, *p* = 0.032).

The hand hit bias marginally shifted towards 0 over training demonstrating an increased use of their left hand, regardless of the laterality index measured at baseline (*H*(2) = 5.250, *p* = 0.072) (Figure 5(c)). There was a significant difference between intervention groups for hand hit bias (*H*(2) < 7.221, *p* < 0.027) at both posttraining and follow-up where the sham group had a smaller bias compared to the HD-tDCS group (*H*(2) = 7.875, *p* = 0.019 and *H*(2) = 7.220, *p* = 0.027, respectively). Baseline left-hand speed correlated with the baseline PPT_L_ score (*p* = 0.407, *r* = 0.048), where faster movements correlated with a higher PPT_L_ score. Left-hand speed did not increase over training (*H*(2) < 3.083, *p* = 0.214) or across intervention groups (all *H*(2) < 0.736, *p* > 0.692). Untrained right-hand speed also did not change over training (*H*(2) = 0.583, *p* = 0.747). There was, however, an overall difference in right-hand speed across intervention groups at posttraining (*χ* ^2^(2) = 6.255, *p* = 0.044) and follow-up (*χ* ^2^(2) = 10.460, *p* = 0.005) where post hoc comparisons identified a significant increase from pre- to posttraining in the HD-tDCS group compared to sham (*χ* ^2^(2) = 2.440, *p* = 0.044). At follow-up, the HD-tDCS group had higher right-hand speed compared to sham and tDCS groups (*χ* ^2^(2) = 3.041, *p* = 0.007 and *χ* ^2^(2) = 2.475, *p* = 0.040, respectively). There was also a significant training effect on hand speed bias at posttraining (*χ* ^2^(2) = 6.155, *p* = 0.046) and follow-up (*χ* ^2^(2) = 6.540, *p* = 0.038) where the HD-tDCS group showed higher hand speed bias at both training points compared to sham (*χ* ^2^(2) = 2.404, *p* = 0.049 and *χ* ^2^(2) = 2.546, *p* = 0.033, respectively).

## 4. Discussion

We quantified changes in sensorimotor function induced by tDCS-enhanced motor learning in children using robotics. We demonstrated coherence between baseline function and improvements in the trained task and multiple robotic measures. Improvements in specific components of visually guided reaching and kinesthesia were observed with motor learning across intervention groups. Intervention-specific effects were not clear. Our findings demonstrate the ability of robotics to explore motor learning and its modulation by noninvasive neurostimulation in children.

Robotics have been a vital tool to understand motor learning in adults including defining specific metrics such as the corrective responses important in motor learning of goal direct reaching [49]. Long-latency responses are important in corrective movements and have been shown to be correlated with reaching errors during learning [50]. Motor learning requires both feedforward and feedback models. Feedforward models utilize state estimation of limb position and are highly involved in goal-directed reaching [51]. Feedback mechanisms are modulated by GABA interneurons in the spinal cord, and ablation of these neurons involved in proprioceptive afferents results in forelimb oscillations in reaching [52]. Robotics can also be utilized as a training tool in motor learning [21]. Robotic applied force fields can alter movement trajectory and assess forces of corrective motions [53]. We believe our results add a novel component to these diverse explorations of motor learning mechanisms by expanding into the realm of noninvasive neuromodulation in pediatrics.

The correlations we observed between KINARM metrics and PPT performance at baseline support the use of this robotic tool in studying the mechanisms of motor learning in children performing this task. The KINARM robot is a validated, well-studied, objective measure of sensorimotor function. Previous studies have demonstrated that the retest reliability of visually guided reaching [27, 48], kinesthesia [54], and position matching [28, 48] tasks is strong. The retest reliability of the object hit may be more variable, as retest reliability is high in adults [46], but a learning effect is seen in pediatric population [48]. To overcome learning effects on the object hit task, we opted to add a practice session at the beginning of the KINARM assessments, which was not included in the analysis. Given the strong retest reliability of our KINARM assessments, we postulate that the sensorimotor changes we observed are not related to a learning effect on the KINARM. Rather, these changes can be attributed to hand dexterity training or a combination of training and intervention.

Robot technologies have also been utilized in tDCS studies in adults and pediatric disease populations. A case study of an adult participant with unilateral spastic CP found that reaching accuracy on a robotic task was improved when conventional anodal tDCS was applied over multiple sessions combined with robotic therapy [55]. Another single session trial of children with CP receiving ipsilesional M1 tDCS or sham, combined with functional training, examined changes in spatiotemporal variables associated with upper arm reaching movement [56]. This study described a reduction in total and returning movement durations in both the paretic and nonparetic limbs in the tDCS group but not the sham controls. Here, we observed that after five days of hand training, overall reaching movements were faster and less corrective movements were made. In our study, only a modest number of specific effects possibly related to tDCS intervention were suggested. This finding may be due to our participants having intact sensorimotor function, where it may be difficult to detect small functional changes. This is in contrast to studies of clinical populations such as CP, which have pronounced sensorimotor deficits that may be more sensitive to change.

Few tDCS motor learning studies have examined sensorimotor functional correlates. One pilot study of five adult stroke or traumatic brain injury participants applied bihemispheric tDCS paired with upper extremity physical therapy and examined effects on PPT scores and the KINARM visually guided reaching and object hit tasks [57]. Findings suggested possible effects on the path length ratio and the miss bias of the object hit task at posttraining compared to baseline. However, the same study did not report changes in PPT scores, which may be due to the study design that focused on gross motor training. Our study identified a marginal shift in hand hit bias across training but not a change in the miss bias over training or intervention. The observed shift in hand hit bias may be attributed to the intensive nondominant hand training that participants underwent, which may have assisted in improving left-hand function. Taken together, there is a clear need to synergize both measurement and training tasks to understand the meaning of alterations in robotic measures of sensorimotor function.

Motor learning is not a unidirectional process but rather requires constant sensory signals to inform the motor system. A controlled study of children and adults with CP performing fine motor training through piano playing for four consecutive weeks demonstrated an improved ability to sense and perceive local vibrations [58]. Another study found that a motor learning paradigm involving velocity-dependent force fields improved proprioceptive estimates of hand position in space [16]. These studies support our findings that hand dexterity training can alter proprioceptive function. The object hit task also required visuospatial attentiveness. We demonstrated improved bimanual motor ability including a possible shift in hand hit bias, where both hands were used more equally. Interestingly, despite showing enhanced motor learning, the HD-tDCS group showed a significant increase in hand hit bias towards the right hand. Whether this finding is due to changes in visuospatial attention or motor function requires further study. As well, these effects suggest that HD-tDCS may have differential effects on sensory motor function, highlighting a need for mechanistic studies in adults and children.

The results obtained here contribute novel data to the growing, but limited, field of noninvasive neuromodulation in children. There has been marked electric field strength differences experienced in the adult and developing brain when tDCS is applied. Electric field differences may be due to skull thickness, CSF volume, and age-dependent differences in grey and white matters [2, 59]. These factors suggest that children experience different electric field patterns compared to adults and may contribute to the differences in observed effects between these populations. Unlike long-standing adult evidence, the proof of principle study which showed that conventional tDCS of the motor cortex can enhance motor learning in healthy school-age children was only recently completed [4]. Also, unlike much of the adult evidence to date, these findings have recently been replicated within the larger trial on which the current study is based, demonstrating that both conventional and HD-tDCS can enhance motor skill acquisition [7]. Safety data for tDCS applications in children is increasingly established but still represents a very small proportion of the published evidence [60, 61]. The KINARM data presented here adds to and further reinforces the favourable safety profile of tDCS in the developing brain by demonstrating no decreases in detailed metrics of sensory and motor functions within a controlled study.

Our study explored possible sensory and motor effects of anodal tDCS and HD-tDCS targeting M1. In addition to motor control, M1 may also be involved in aspects of proprioception, such as position sense and kinesthesia. Neuroimaging studies have demonstrated activations during kinesthesia-related tasks in the Brodmann areas 4a, 4p, and 6 and supplementary motor area during kinesthetic illusions [62]. Brodmann area 2, which is more classically involved in kinesthesia, was also activated. Such studies have demonstrated that kinesthesia is associated with motor areas, and robust connections exist throughout the frontal and parietal cortices, all of which may have been influenced by tDCS and, less likely, by HD-tDCS. Our inability to identify any large effects of tDCS or HD-tDCS on kinesthesia may relate to multiple factors.

Our study examined correlations between baseline KINARM sensorimotor function across age groups and motor function. In both healthy control and stroke-affected adults, visually guided reaching and position matching task variables have been correlated with PPT [17]. In healthy children, however, the PPT score may not correlate with Var_xy_ or Shift_xy_ [31], suggesting age-specific differences. This previous finding is inconsistent with our work, where we showed that baseline Var_xy_ was significantly correlated with baseline PPT_L_. We identified a correlation between baseline reaction time and baseline PPT_L_, with no correlation to the remaining variables. A pediatric study of healthy controls and concussion patients found that nondominant hand PPT scores correlated with reaction time, initial distance ratio, and path length ratio in the visually guided reaching task [63]. This study also reported a correlation between nondominant hand PPT scores and hits with their nondominant hand in the object hit task. We did not identify such a correlation with nondominant hand PPT scores and baseline total hits with the left hand. Therefore, our study was able to reproduce some but not all correlations of previous studies.

Previous work by our lab suggested that perinatal stroke populations show dysfunction in position sense, kinesthesia, and visually guided reaching [31–33]. The application of tDCS paired with motor therapy as a possible treatment to improve sensorimotor function in this clinical population was supported by two early clinical trials [38, 41]. The addition of detailed behavioral outcomes has been suggested as an important outcome in the design of such childhood disability trials as they move forward [37]. Our results here support the feasibility of this approach while also helping to define the potential and limitations of the ability of such measures to demonstrate intervention-induced change.

Our study has important limitations. Our ability to fully define the effects of tDCS and HD-tDCS on robotic outcomes may have been restricted by our modest sample size, which was powered on the primary clinical outcome [7]. Our age range spans a group of children and adolescents that are developmentally unique, possibly contributing to substantial variability in response. Our study excluded younger children, who are also in need of investigation. The differential effects of tDCS on various age groups are poorly understood. We had a comprehensive and consistent protocol with multiple set breaks to minimize fatigue effects, but these cannot be excluded and likely varied across subjects. There are other known factors that may dictate responsiveness to brain stimulation that we could not control including sleep, experience, and genetics.

In conclusion, robotics can quantify task-specific sensorimotor functions before and after motor training and neurostimulation interventions in children. Hand motor training may be mediated by specific improvements in elements of visually guided reaching and kinesthesia. Although tDCS and HD-tDCS can enhance such motor learning, the robotic sensorimotor correlates of such neuromodulation may require more powerful studies to be defined.

## Figures and Tables

**Table 1:** 

Stimulation group	Age (SD)	Laterality index (SD)	Sex F : M	Baseline PPT_L_ (SD)	Baseline PPT_R_ (SD)	Baseline PPT_L_ vs. PPT_R_
Sham	15.8 (1.3)	81.9 (22.8)	3 : 5	13.8 (1.3)	15.2 (1.9)	*p* = 0.013
tDCS	15.9 (1.5)	82.5 (13.1)	6 : 2	13.5 (1.3)	15.2 (1.9)	*p* = 0.011
HD-tDCS	14.8 (2.0)	81.3 (14.6)	4 : 4	13.9 (1.9)	15.8 (1.6)	*p* < 0.001
Mean	15.5 (1.7)	81.9 (16.6)	13 : 11	13.8 (1.5)	15.4 (1.7)	*p* < 0.001
Between groups	*p* = 0.324	*p* = 0.879	*p* = 0.309	*p* = 0.846	*p* = 0.741	—

Age = age in years at enrollment; laterality index measured through the modified Edinburgh handedness inventory; baseline PPT_L_ = left-hand Purdue pegboard score measured at baseline; baseline PPT_R_ = right-hand Purdue pegboard score measured at baseline.

## Data Availability

The data used to support the findings of this study are included within the article and available from the corresponding author upon request.

## Supplementary Materials

Supplementary Materials Supplementary Figure 1: trial design. tDCS = transcranial direct current stimulation; HD-tDCS = high-definition transcranial direct current stimulation; PPT = Purdue pegboard test. Supplementary Figure 2: electrode montages. (a) Electrode montages for the sham and tDCS group. The red sponge electrode demonstrates the anode, and the blue sponge electrode indicates the cathode. (b) HD-tDCS montage. The red circle demonstrates the anode, and blue circles indicate the cathodes.
