# Supplementary figures and images for: Sensorimotor Robotic Measures of tDCS- and HD-tDCS-Enhanced Motor Learning in Children
Source: Neural Plast. 2018 Dec 18;2018:5317405. doi: 10.1155/2018/5317405 (PMC6312578; doi:10.1155/2018/5317405)

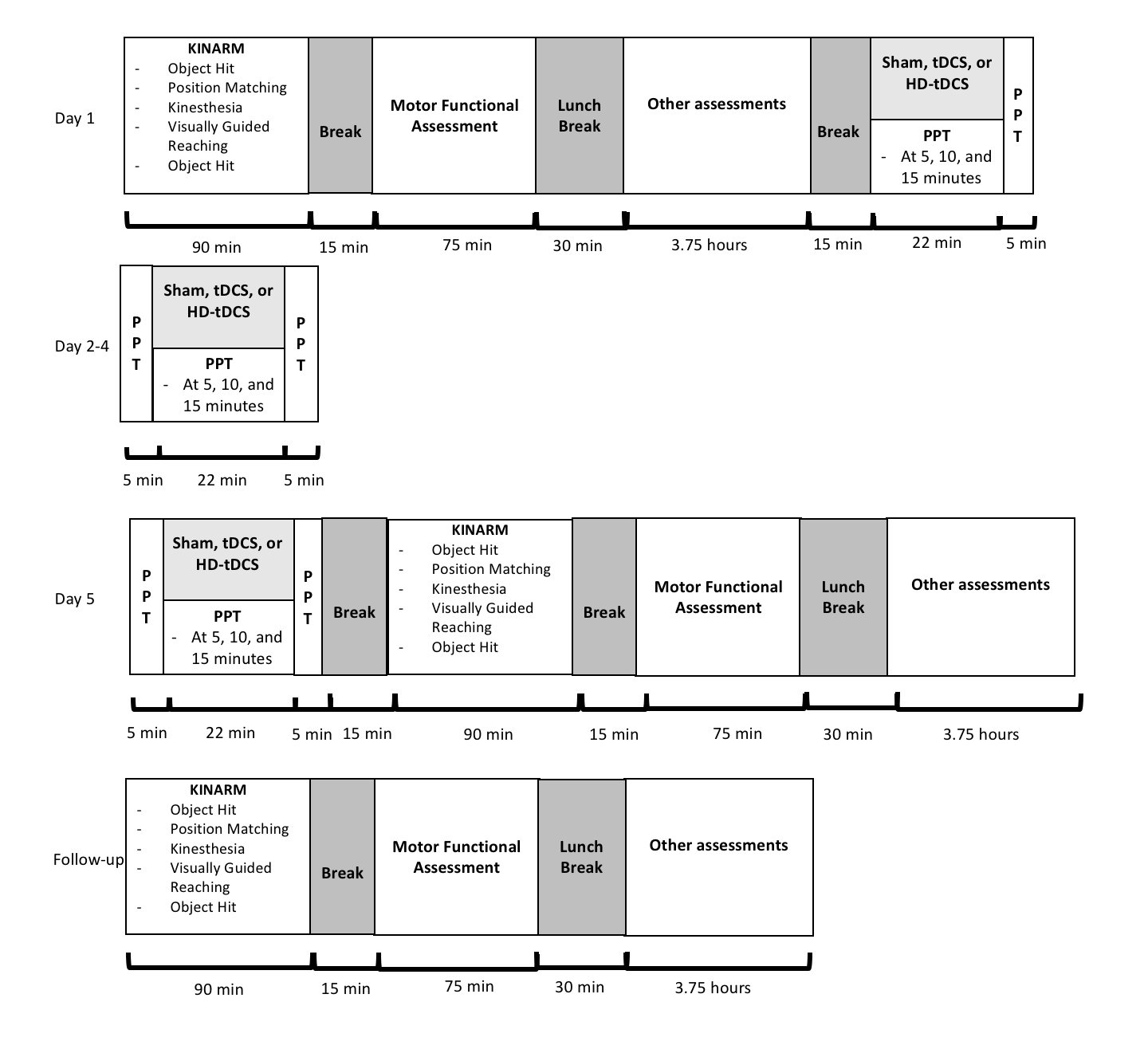

Supplement: Supplementary Materials — Supplementary Figure 1: trial design. tDCS = transcranial direct current stimulation; HD-tDCS = high-definition transcranial direct current stimulation; PPT = Purdue pegboard test. Supplementary Figure 2: electrode montages. (a) Electrode montages for the sham and tDCS group. The red sponge electrode demonstrates the anode, and the blue sponge electrode indicates the cathode. (b) HD-tDCS montage. The red circle demonstrates the anode, and blue circles indicate the cathodes. [file 5317405.f1.zip › Supplementary Figure 1.png]

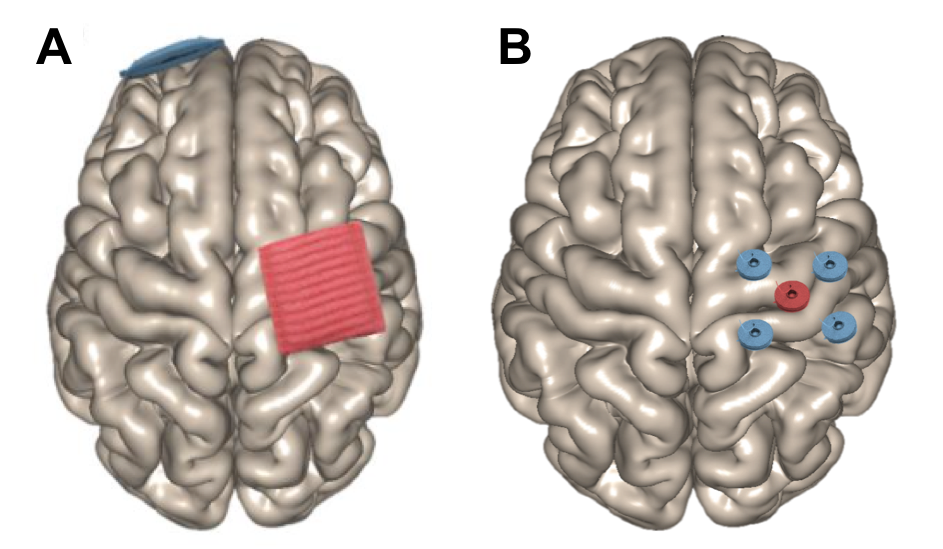

Supplement: Supplementary Materials — Supplementary Figure 1: trial design. tDCS = transcranial direct current stimulation; HD-tDCS = high-definition transcranial direct current stimulation; PPT = Purdue pegboard test. Supplementary Figure 2: electrode montages. (a) Electrode montages for the sham and tDCS group. The red sponge electrode demonstrates the anode, and the blue sponge electrode indicates the cathode. (b) HD-tDCS montage. The red circle demonstrates the anode, and blue circles indicate the cathodes. [file 5317405.f1.zip › Supplementary Figure 2.png]
